# Association between dynapenic obesity phenotypes and physical performance in middle-age and older women living in community

**DOI:** 10.3389/fnut.2024.1480284

**Published:** 2024-09-25

**Authors:** Cecilia Arteaga-Pazmiño, Diana Fonseca-Pérez, Manuel Balladares Mazzini, Javier Galvez-Celi, Janet Emén Sánchez, Ludwig Álvarez-Córdova

**Affiliations:** ^1^Carrera de Nutrición y Dietética, Facultad de Ciencias Médicas, Universidad de Guayaquil, Guayaquil, Ecuador; ^2^Carrera de Nutrición y Dietética, Facultad de Ciencias de la Salud, Universidad Católica de Santiago de Guayaquil, Guayaquil, Ecuador; ^3^Carrera de Medicina, Facultad de Ciencias Médicas, Universidad de Guayaquil, Guayaquil, Ecuador; ^4^Maestría de Nutrición y Dietética, Facultad de Ciencias de la Salud, Universidad de Las Américas (UDLA), Quito, Ecuador

**Keywords:** dynapenic obesity, physical performance, middle-age women, community-dwelling, dynapenia

## Abstract

**Background:**

Dynapenic obesity (DO) is the coexistence of excess adipose tissue/body weight and low muscle strength. This condition is associated with an increased risk of suffering from various chronic diseases and physical deterioration in older people.

**Aim:**

To analyze the association between DO phenotypes and physical performance in middle-aged women living in the community.

**Methods:**

This cross-sectional study was conducted on middle-aged and older women (≥50 years) residing in Guayaquil, Ecuador. Dynapenia was diagnosticated by a handgrip strength (HGS) < 16 kg; obesity was determined based on body mass index (BMI) ≥ 30 kg/m^2^. Participants were categorized into four groups based on their dynapenia and obesity status: non-dynapenic/non-obesity (ND/NO), obesity/non-dynapenic (O/ND), dynapenic/non-obesity (D/NO) and dynapenic/obesity (D/O). Physical performance was assessed by the Short Physical Performance Battery (SPPB).

**Results:**

A total of 171 women were assessed. The median (IQR) age of the sample was 72.0 (17.0) years. Obesity and dynapenia were 35% (*n* = 60) and 57.8% (*n* = 99) of the participants, respectively. The prevalence of ND/NO was 25.1% (*n* = 43), O/ND 17% (*n* = 29), D/NO 39.8% (*n* = 68) and DO 18.1% (*n* = 31). The mean SPPB total score was 6.5 ± 3.2. Participants of D/NO and DO groups presented significantly lower mean SPPB scores (*p* < 0.001) compared to those of NO/ND and O/ND groups.

**Conclusion:**

Women with DO and D/NO exhibited significantly lower SPPB scores, indicating poorer physical performance. These findings emphasize the importance of incorporating a comprehensive assessment of muscle strength and obesity in middle-aged and older women.

## 1 Introduction

Obesity is a multifactorial, chronic, progressive disease associated with adverse health outcomes throughout the life course (1, 2). In 2022, an estimated 374 million women were identified with obesity (3), however, data on the prevalence of obesity specifically in women aged 50 and older is lacking.

In middle-aged women, several factors contribute to changes in body composition. These include age-related decline in estrogen levels around menopause (4, 5) and its impact on metabolism and related diseases (6, 7), lifestyle factors (8–10) such as diet (11), anabolic resistance associated with aging (12), among others. As a result, decreasing of muscle mass and strength, which begin to decrease around 30 and accelerate after 40 (13, 14), infiltration of fat within muscle and increasing prevalence of dynapenia (weakness) (15), sarcopenia (weakness and muscle loss), and obesity are common in this aged group (16).

Moreover, a wide range of alterations, including altered immune function, increased systemic inflammation, accumulated intracellular macromolecules, decreased genomic integrity, and changes in tissue and body composition (17), are common to both obesity and aging (18, 19).

In the last few years, the concept of dynapenic obesity (DO) has been used to describe the coexistence of excess adipose tissue/body weight and low muscle strength (20). Different criteria have been used to identify the obesity component, such as body mass index (BMI) (21), abdominal obesity (22), and fat mass percentage (23). DO is not a homogenous condition and different phenotypes might exist based on variations in factors like fat distribution and muscle quality.

Regardless of the criteria to identify obesity, DO has been associated with a higher risk of falls (24), poorer bone health (25), inflammatory biomarkers (26), and an increased risk of chronic diseases (27). Given the independent effect of obesity on muscle function (28, 29), DO could be associated with worse physical performance. In individuals with obesity have been reported impaired functional capacity (30); particularly, women with obesity exhibited slower fast gait speeds, shorter stride lengths, poorer sit-to-stand performance, and endurance (31). Nevertheless, high handgrip strength levels could attenuate the negative effect of adiposity (32).

Moreover, recent studies on the association between DO and physical performance in middle-aged women and older show conflicting results (33, 34), which might be due to population characteristics and heterogeneity in DO definitions. We previously reported the prevalence of sarcopenia and obesity in community-dwelling older adults (35), however, the current prevalence of DO in middle-aged and older women remains unknown.

Understanding different DO phenotypes can provide more specific insights into the relationship with physical performance and ultimately lead to more targeted interventions. Thus, this study aimed to assess the relationship between DO phenotypes and physical performance in middle-aged women living in the community.

## 2 Materials and methods

### 2.1 Subjects

**Figure 1:**
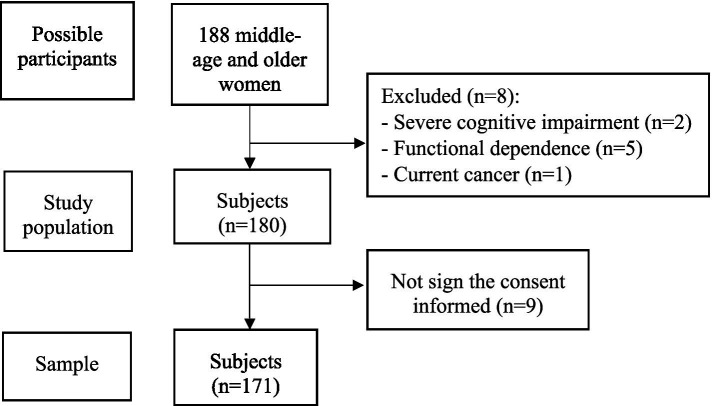
Flowchart of the recruitment process of the participants of the study.

This was an observational cross-sectional study carried out in community-dwelling, middle-aged and older women living in urban-marginal areas of Guayaquil, Ecuador from November 2019 to December 2020. The following criteria were used for inclusion: women in the ≥50 years old who agreed to participate voluntarily in the study signing an informant consent. The exclusion criteria were institutionalized individuals, those with known dementia or severe cognitive impairment, functional dependence, current cancer, chronic obstructive pulmonary disease, and musculoskeletal diseases. Figure 1 shows the sample selection flowchart.

### 2.2 Sociodemographic and clinical characteristics

Participants filled out a self-reported survey with a standardized questionnaire that assessed their socioeconomic and clinical characteristics. Socioeconomic variables include: age, ethnicity (mestizo, afro-Ecuadorian, Caucasian, indigenous), marital status (single, married, widowed, divorced), education level (none, primary, secondary, tertiary). Clinical characteristics were assessment by prevalent medical conditions such as type 2 diabetes, hypertension, dyslipidemia, gastroesophageal reflux disease, arthritis, constipation.

### 2.3 Dynapenia measurement

Dynapenia was diagnosticated by handgrip strength (HGS) using a Jamar Plus Hand Dynamometer with an accuracy of over 99% (36). HGS was evaluated in both hands, regardless of the dominant one. Subjects were advised verbally to grip the instrument and perform maximum handgrip strength. All the lectures were carried out standing, with both arms pending sideways and the dynamometer facing the evaluator. The value registered was the higher value realized by side, individuals rest 1 min at least between trials of the same hand. Dynapenia was evaluated by handgrip strength defined as HGS < 16 kg according to the European Working Group on Sarcopenia in Older People (EWGSOP2) (37).

### 2.4 Obesity measurement

Obesity was identified according to body mass index (BMI), calculated as weight in kilograms divided by height in meters squared (kg/m^2^). Body mass (weight) was measured on a SECA 700 ® mechanical physical scale and recorded in kilograms (kg) to the nearest 0.1 decimal. Height was recorded on a SECA 213® portable stadiometer. Obesity was determined based on BMI ≥ 30 kg/m^2^ (38).

### 2.5 Dynapenic obesity phenotypes

Participants were categorized into four groups based on their dynapenia and obesity status: non-dynapenic/non-obesity (ND/NO), obesity/non-dynapenic (O/ND), dynapenic/non-obesity (D/NO) and dynapenic/obesity (D/O) (39).

### 2.6 Physical performance measurement

The Short Physical Performance Battery (SPPB) was used to assess physical performance. The SPPB comprises three physical performance measures: standing balance, repeated chair stands, and gait speed (40). Evaluation of balance involved hierarchical tasks consisting of side-by-side, semi-tandem, and full-tandem stands. During the repeated chair stand test, participants underwent timing while performing five sit-to-stand repetitions. Gait speed assessment was conducted by timing participants as they walked 2.44 meters at their usual pace.

Each assessment is graded on a scale ranging from 0 (indicating an inability to complete the task) to 4 points (representing the highest level of performance) on the test. The overall score for the SPPB falls within the range of 0 (indicating the poorest performance) to 12 points (indicating the best performance) and assesses performance in the various tests based on three or four distinct categories of scores: three categories include 0–6 points (indicating subpar performance), 7–9 points (indicating moderate performance), and 10–12 points (indicating good performance); while four categories consist of 0–3 points (indicating disability/very poor performance), 4–6 points (indicating poor performance), 7–9 points (indicating moderate performance), and 10–12 points (indicating good performance).

### 2.7 Other variables

The following body composition compartments were also measured, using a Multifrequency Segmental Body Composition Analyzer (InBody 270 DSM-BIA®): muscle mass, fat mass, and body fat percentage, as well as their index. To assess BC, participants were advised not to eat or drink 4 h before the test, consume any caffeine beverage or alcohol within 12 h of the test, use diuretic medication, perform exercise 12 h before the test, and suggest evacuating urine.

### 2.8 Ethical considerations

The study’s approval came from the Ethics Committee for Research in Humans of the “Hospital Clínica Kennedy,” Guayaquil, Ecuador (CEISH No: HCK-CEISH-19-0038, June 21, 2019) and conducted by the guidelines of the Declaration of Helsinki. All participants were informed of the study, its aims, and used instruments, following which they gave written permission to take part.

### 2.9 Data analysis

Data analysis was performed using IBM SPSS Statistics (version 25.0; IBM, Chicago, IL, EE. UU). Study participants were divided into groups according to DO phenotypes. Continuous variables are reported as mean and standard deviation or median and interquartile range (IQR) in the descriptive analysis, and categorical variables as frequencies and percentages. For the bivariate analysis, the numerical variables with normal distribution were compared using the Anova test; contrary to this, we used the Kruskall-Wallis test. For all analyses, a *p* value <0.05 was considered statistically significant.

## 3 Results

A total of 171 middle-aged and older women participated in this study. The median (IQR) age of the sample was 72.0 years (17.0). Obesity and dynapenia were 35.1% (*n* = 60) and 57.8% (*n* = 99) of the participants, respectively. The prevalence of ND/NO was 25.1% (*n* = 43), O/ND 17% (*n* = 29), D/NO 39.8% (*n* = 68) and DO 18.1% (*n* = 31). Subjects with D/NO were older compared with other phenotypes (*p* < 0.001).

Participants with DO had a higher BMI, waist circumference, fat mass index, and visceral fat compared with the other phenotypes. HGS and phase angel were higher in those with NO/ND and O/ND, compared with the other phenotypes, while skeletal muscle mass was higher in participants with O/ND and D/O phenotypes in contract with others groups. The sociodemographic and clinical characteristics of the participants, according to DO phenotypes, are presented in Table 1.

**Table 1:** Characteristics of the studied population according to dynapenic obesity phenotypes.

Variable	Total (*n* = 171)	NO/ND (*n* = 43)	O/ND (*n* = 29)	D/NO (*n* = 68)	D/O (*n* = 31)	*p*-value
Age (years)	72 (17.0)	72.0 ± 9.8	65.4 ± 9.3	77.4 ± 10.4	70.0 ± 12.7	< 0.001*
Ethnicity, *n* (%)						
Mestizo	119 (69.6)	24 (55.8)	24 (82.7)	47 (69.1)	24 (77.4)	0.136
Afro-Ecuadorian	24 (14)	10 (22.3)	5 (17.2)	6 (8.8)	3 (9.6)	
Caucasian	15 (8.7)	5 (11.6)	0 (0.0)	8 (11.8)	2 (6.4)	
Indigenous	13 (7.6)	4 (9.3)	0 (0)	7 (10.3)	2 (6.4)	
Marital status, *n* (%)						
Single	48 (28.1)	13 (30.2)	9 (31.0)	20 (29.4)	6 (19.3)	0.147
Married	58 (33.9)	14 (32.5)	11 (37.9)	16 (23.5)	17 (54.8)	
Widowed	48 (28.01)	12 (27.9)	6 (20.7)	26 (38.2)	4 (12.9)	
Divorced	17 (9.9)	4 (9.30)	3 (10.3)	6 (8.8)	4 (12.9)	
Education level, *n* (%)						
None	45 (26.3)	10 (23.2)	9 (31.0)	19 (27.9)	7 (22.6)	0.914
Primary	94 (54.9)	25 (58.1)	14 (48.2)	38 (55.9)	17 (54.8)	
Secondary	27 (15.8)	8 (18.6)	5 (17.2)	8 (11.8)	6 (19.3)	
Tertiary	5 (2.9)	0 (0)	1 (3.4)	3 (4.4)	1 (1.4)	
Medical conditions, *n* (%)						
None	13 (7.6)	3 (6.9)	2 (6.8)	7 (10.2)	1 (3.2)	0.468
T2D	25 (14.6)	2 (4.6)	4 (13.7)	14 (20.6)	5 (16.1)	
Hypertension	94 (54.9)	26 (60.5)	19 (65.5)	30 (44.1)	19 (61.2)	
Dyslipidemia	10 (5.8)	3 (6.9)	1 (3.4)	5 (7.3)	1 (3.2)	
GERD	2 (1.2)	1 (2.3)	0 (0)	1 (1.4)	0 (0)	
Arthritis	12 (7)	1 (2.3)	1 (3.4)	7 (10.2)	3 (9.6)	
Constipation	15 (8.7)	7 (16.2)	2 (6.8)	4 (5.9)	2 (6.4)	
Weight (kg), Median (IQR)	62 (53–71)	61 (55–66)	75 (68–82)	52.9 (45.8–60)	73 (66–86.1)	< 0.001*
Height (cm)	148 (143–153)	150 (145.5–156.9)	150 (143.5–153.7)	146.4 (141.1–151)	148.6 (144–151)	0.006*
BIM (kg/m^2^)	28.4 ± 5.3	26.1 ± 2.6	34.0 ± 3.0	24.8 ± 3.5	34.5 ± 3.2	< 0.001*
Waist circumference (cm)	94.0 ± 11.7	90.2 ± 8.5	102.6 ± 7.8	87.7 ± 10.1	104.7 ± 9.6	< 0.001*
Skeletal muscle mass (kg)	15 (11.6–18.4)	14.4 (12.7–16.6)	18.9 (15.9–21.3)	12 (8.8–15.7)	18.6 (14.8–21.3)	< 0.001*
Fat mas index (kg/m^2^)	11.9 (10.1–15)	11.6 (10.1–12.9)	15.6 (14–17.1)	10.5 (7.8–12)	15.7 (13.4–18.6)	< 0.001*
Visceral fat (%)	2.9 (2.4–3.5)	2.7 (2.4–3.1)	3.3 (2.7–3.7)	2.6 (2.2–3)	3.8 (3.1–4.2)	< 0.001*
Phase angle	5.2 (4.6–5.9)	5.2 (4.6–5.5)	5.7 (4.9–6.1)	4.8 (4.2–5.7)	5.7 (5–6.9)	< 0.001*
HGS (kg)	14 (10–19)	20 (18–22)	20 (17–25)	11.5 (10–12)	12 (10–12)	< 0.001*

^*^p value < 0.05.

Continuous symmetric variables are presented as means ± SD or median (IQR) for asymmetric variables, or numbers (percentages) for categorical variables unless otherwise indicated.

ND/NO, non-dynapenic/non-obesity; O/ND, obesity/non-dynapenic; D/NO, dynapenic/non-obesity; D/O, dynapenic/obesity; IQR, interquartile range; SD, standard deviation; T2D, type 2 diabetes; GERD, gastroesophageal reflux disease; HGS, handgrip strength.

**Figure 2:**
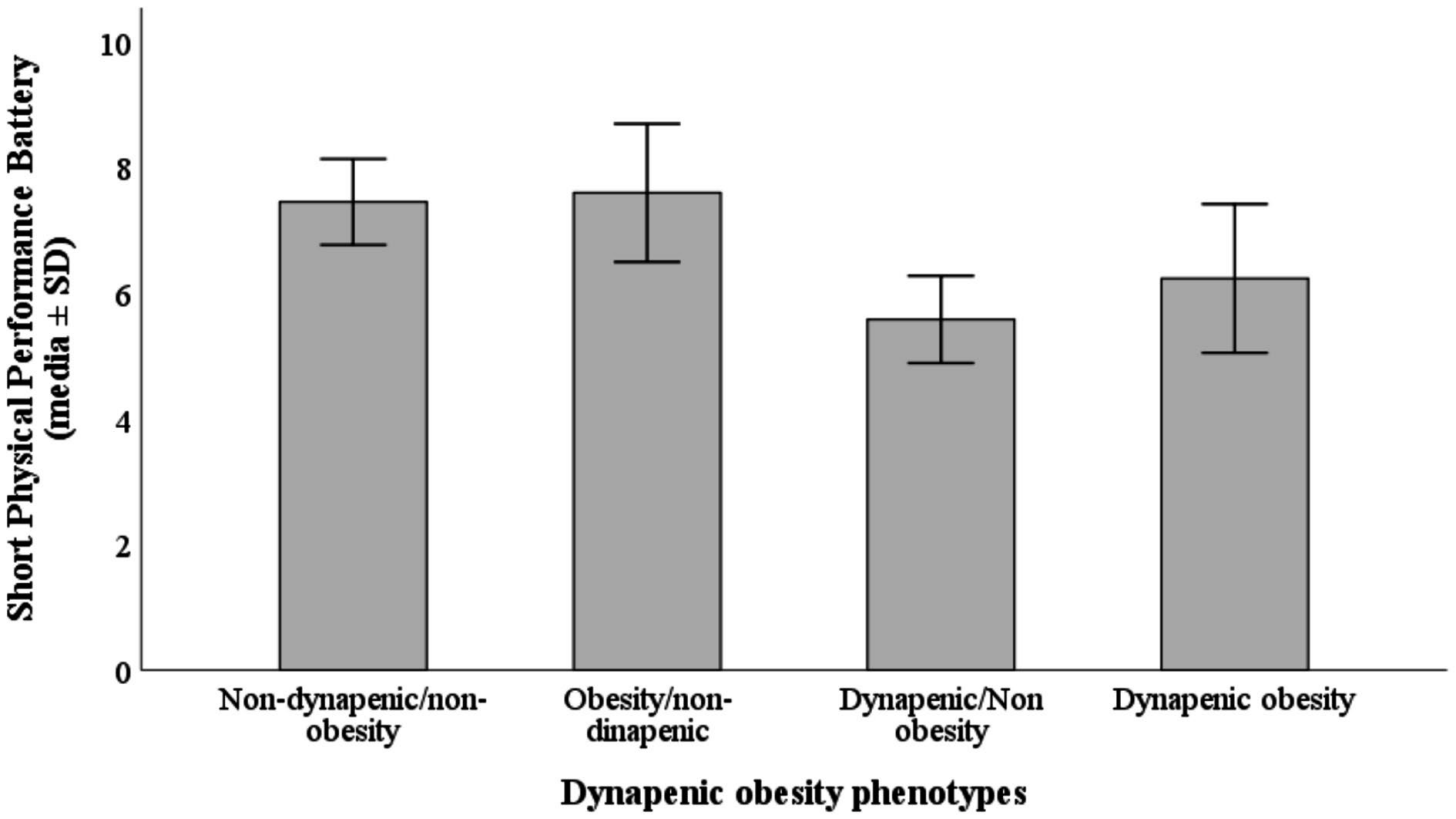
Mean Short Physical Performance Battery (SPPB) total score according to dynapenic obesity phenotypes.

The mean SPPB total score was 6.5 ± 3.2 Participants of D/NO and DO groups presented significantly lower mean SPPB scores (*p* < 0.001) compared to those of NO/ND and O/ND groups (Figure 2).

Very poor performance was prevalent in 22.6% (*n* = 7), while poor performance, moderate performance, and good performance were prevalent in 16.1% (*n* = 5), 54.8% (*n* = 17) and 6.4% (*n* = 2) in the sample, respectively (Table 2).

**Table 2:** Characteristics of the studied population according to dynapenic obesity phenotypes.

Physical performance	Total (*n* = 171)	NO/ND (*n* = 43)	O/ND (*n* = 29)	D/NO (*n* = 68)	D/O (*n* = 31)	*p*-value
SPPB value, Media ± SD	6.5 ± 3.2	7.4 ± 2.2	7.5 ± 2.9	5.5 ± 2.7	6.2 ± 3.9	0.001*
Very poor performance	28 (16.3)	1 (2.3)	2 (6.8)	18 (26.4)	7 (22.6)	0.004*
Poor performance	45 (26.3)	12 (27.9)	7 (24.1)	21 (30.9)	5 (16.1)	
Moderate performance	80 (46.7)	24 (55.8)	13 (44.8)	26 (38.2)	17 (54.8)	
Good performance	18 (10.5)	6 (13.9)	7 (24.1)	3 (4.4)	2 (6.4)	

^*^p value < 0.05.

ND/NO, non-dynapenic/non-obesity; O/ND, obesity/non-dynapenic; D/NO, dynapenic/non-obesity; D/O, dynapenic obesity; SPPB, Short Physical Performance Battery; SD, standard deviation.

## 4 Discussion

This report aims to enhance understanding of the phenotypes of dynapenia, obesity, and DO in middle-aged and older women living in the community and highlight the detrimental effect of DO on physical function, exceeding the negative effects of either phenotype alone.

To our knowledge, the only report on the prevalence of muscle weakness in older adults was published by Garces, based on the data from the first National Health, Wellbeing, and Aging Survey (21). Later, he reported a lower prevalence of 6.8% phenotype of DO in female older adults, in comparison to 18.1% in our data. This result can be related to a more representative sample size, in contrast with our population of mostly urban-marginal middle-aged women. In other variables, the prevalence of obesity was 35.1% vs. 20%, and the prevalence of only dynapenia was significantly higher in our data with 57.8% vs. 24.7% (39).

Our main findings showed that the D/NO phenotype had the worse scores for physical performance in middle-aged women and older women in the SPPB test, followed by the DO group; we found statistical differences in the SPPB value for the four categories. Anthropometric characteristics of the population related to an increase in fat mass present statistical differences in the four phenotypic groups weight, height, BMI, waist circumference, and visceral fat. Interestingly, skeletal muscle mass was higher in participants with the O/ND phenotype compared to both DO and NO/ND groups. This suggests potential differences in body composition within dynapenic individuals.

Some reports DO have poorer physical function than individuals with obesity alone or dynapenia alone, suggesting a possible independent effect on physical performance measurements, and probably these effects are considered additive and not multiplicative (34, 41). Furthermore, based on cross-sectional and longitudinal studies that have described the mixture effect of obesity and poor muscle strength in older adults, defined as DO, this condition increases the probability of mobility disability, poor functional performance, risk of falls, hospitalization, and higher mortality (41, 42).

Low muscle mass function and obesity affect more than one in ten older adults globally (43). Our data shows that the prevalence of DO in our sample was 18.1%. Stenholm et al. evaluated 930 adults aged 65 and older in a 6-year follow-up period; obesity (cataloged with BMI), and low muscle strength (measured with knee extensor strength) registered a 17% reduction of walking speed, in comparison with 8% counterparts with only obesity and 4% individual with lower strength (44). In another study, with 2,208 adults aged 55 years and older, had been described a prevalence of walking limitations significantly higher 61% than their previous reports when DO was diagnosed (45). Additionally, a recent research suggests that diminished gait speed, an indicator of physical performance, can predict a risk of DO (46).

Regardless the relationship between muscle strength and adiposity is related to the determination of the method to diagnose body fat excess. Reports from Keevil et al. show that a larger BMI was associated with lower HGS, but a high waist circumference value has an opposite association. In addition, they found that a greater value waist circumference HGS was lower in both sexes. These findings proposed that abdominal fat is the most metabolically active tissue with the understanding potential mechanism for the association between skeletal muscle and fat mass (47).

Finally, finding obesity phenotypes (48) could help researchers better understand how DO interacts with physical performance, which will advance the study of DO (49).

This study provides valuable insights into sarcopenia (DO) phenotypes in middle-aged and older women residing in the community. When compared to national reference data (39), our findings reveal a significant increase in the prevalence of obesity, dynapenia, and sarcopenia. This highlights the critical need for public health programs and interventions to prevent and address these conditions. The heightened prevalence of sarcopenia emphasizes the need for further research aimed at identifying associated factors and developing strategies to improve muscle health and physical function in this population.

At the national level, the high prevalence of dynapenia and obesity calls for a comprehensive approach to assessment and intervention. Potential strategies could include programs that promote physical activity through public awareness campaigns, community-based exercise and nutrition initiatives, and training healthcare professionals to manage these conditions effectively. Ensuring equitable access to care will require addressing socioeconomic disparities and improving healthcare accessibility across all sectors.

One key limitation of this study is its cross-sectional design, which does not allow for establishing causal relationships between the variables examined (e.g., obesity and dynapenia with physical performance). Furthermore, the study only included middle-aged and older women, limiting the generalizability of the results to younger populations.

## 5 Conclusion

In conclusion, the D/NO and D/O groups presented the worst scores in physical performance and were associated with impaired physical function. The DO group had the highest body fat percentage and worst performance on the SPPB. This suggests the DO phenotype is associated with poorer physical health. This link between the DO phenotype and functional limitations is a key finding that can help establish personalized therapeutic strategies to address the coexistence of these health problems.

## Data Availability

The raw data supporting the conclusions of this article will be made available by the authors, without undue reservation.
